# Internal Mammary Vein Valves: A Histological Study

**DOI:** 10.1038/s41598-020-65810-7

**Published:** 2020-06-01

**Authors:** Yoshitaka Kubota, Yoshihisa Yamaji, Kentaro Kosaka, Hideki Tokumoto, Takafumi Tezuka, Shinsuke Akita, Motone Kuriyama, Nobuyuki Mitsukawa

**Affiliations:** 1 Department of Plastic Surgery, Chiba University 1-8-1, Inohana, Chuo-ku, Chiba-city, Chiba #260-8677 Japan; 2 Department of Plastic Surgery, Maebashi Red Cross Hospital 389-1, Asakura-cho, Maebashi-city, Gunma #371-0811 Japan; 3 Department of Plastic Surgery, Chiba Cancer Center 666-2, Nitona-cho, Chuo-ku, Chiba-city, Chiba #260-8717 Japan; 4 Department of Plastic Surgery, Shin-Yurigaoka General Hospital 255, Aza-Tsuko, Furusawa, Asao-ku, Kawasaki-city, Kanagawa #215-0026 Japan

**Keywords:** Breast cancer, Peripheral vascular disease, Thromboembolism

## Abstract

It is widely accepted that the internal mammary vein (IMV) is valveless. However, few anatomical studies are available on the presence or absence of IMV valves. To test the hypothesis that the IMV is valveless, we performed microscopic histological examination of the IMV. IMV samples were collected from 10 human fresh frozen cadavers. For a control, the small saphenous vein (SSV) was obtained. Histological stains were performed. Microscopic examination showed that a venous valve was found in 8 of 20 IMVs. The structure of the valve leaflet consisted of two parts. There was a “thick part” located near the wall of the vein that consisted of smooth muscle cells and fibers. There was also a “thin part” located near the center of the venous lumen that lacked smooth muscle cells. The size of the thick part of the IMV valve was smaller than the SSV valve, whereas there was no difference in the size of the thin part between the IMV and SSV. IMV valves exist. Our results that an IMV valve was present in less than half of IMVs and there was a small-sized valve leaflet suggest that the IMV valve may be rudimentary.

## Introduction

The internal mammary vein (IMV) is frequently used as a recipient vessel for microvascular anastomosis in breast reconstructive surgery after mastectomy for breast cancer. Autologous abdominal subcutaneous fat tissue is commonly used as a donor tissue. The donor tissue was disconnected from the abdomen, then reconnected to the chest. This technique is called a “free flap.” Using the free flap technique, the donor vessels are microscopically anastomosed to the recipient vessels. One donor artery is anastomosed to one recipient artery because there exists only one artery in each donor and recipient^1^. In contrast, there are one or two IMVs accompanying the internal mammary arteries and there are one or two donor veins accompanying the donor artery.

Retrograde anastomosis to the IMV is used in cases where there is only one IMV and two donor veins^2^. In such cases, one donor vein (first donor vein) is anastomosed to the superior end of the IMV, and the other donor vein (second donor vein) is anastomosed to the inferior end of the IMV. It is expected that venous blood from the second donor vein will flow into the inferior limb of the IMV in a retrograde fashion based on the pressure difference between the high-pressure donor vein and the low-pressure IMV. The valveless IMV hypothesis is important since it suggests that there will be no hindrance of retrograde venous blood flow to the inferior limb of the IMV. A number of clinical reports suggest that the IMV is valveless, so retrograde anastomosis to the IMV should be safe^2–6^.

However, few anatomical studies are available regarding the presence or absence of IMV valves. Mackey *et al*. refuted the valveless IMV hypothesis, showing counter evidence that there were valves in the IMVs of formalin-fixed human cadavers^7^. However, subsequent clinical reports by other authors kept adopting the valveless IMV hypothesis.

To test the hypothesis that the IMV is valveless, we performed a histological study of IMVs from human fresh frozen cadavers.

## Materials and Methods

### Ethics and cadavers

The current study was conducted in accordance with the Ethical Committee of Chiba University School of Medicine (Chiba, Japan [approval numbers 1672 and 3193]). All studies were performed according to the guidelines of the Declaration of Helsinki. Fresh frozen cadavers were obtained from the Clinical Anatomy Lab of Chiba University. Cadavers were obtained and all studies using cadavers were performed according to the Guidelines for Cadaver Dissection in Education and Research of Clinical Medicine promulgated by the Japan Surgical Society and Japanese Association of Anatomists^8^. Written informed consent for scientific research and use of the cadavers was obtained from all donors or authorized representatives.

### IMV samples and histologic staining

**Figure 1:**
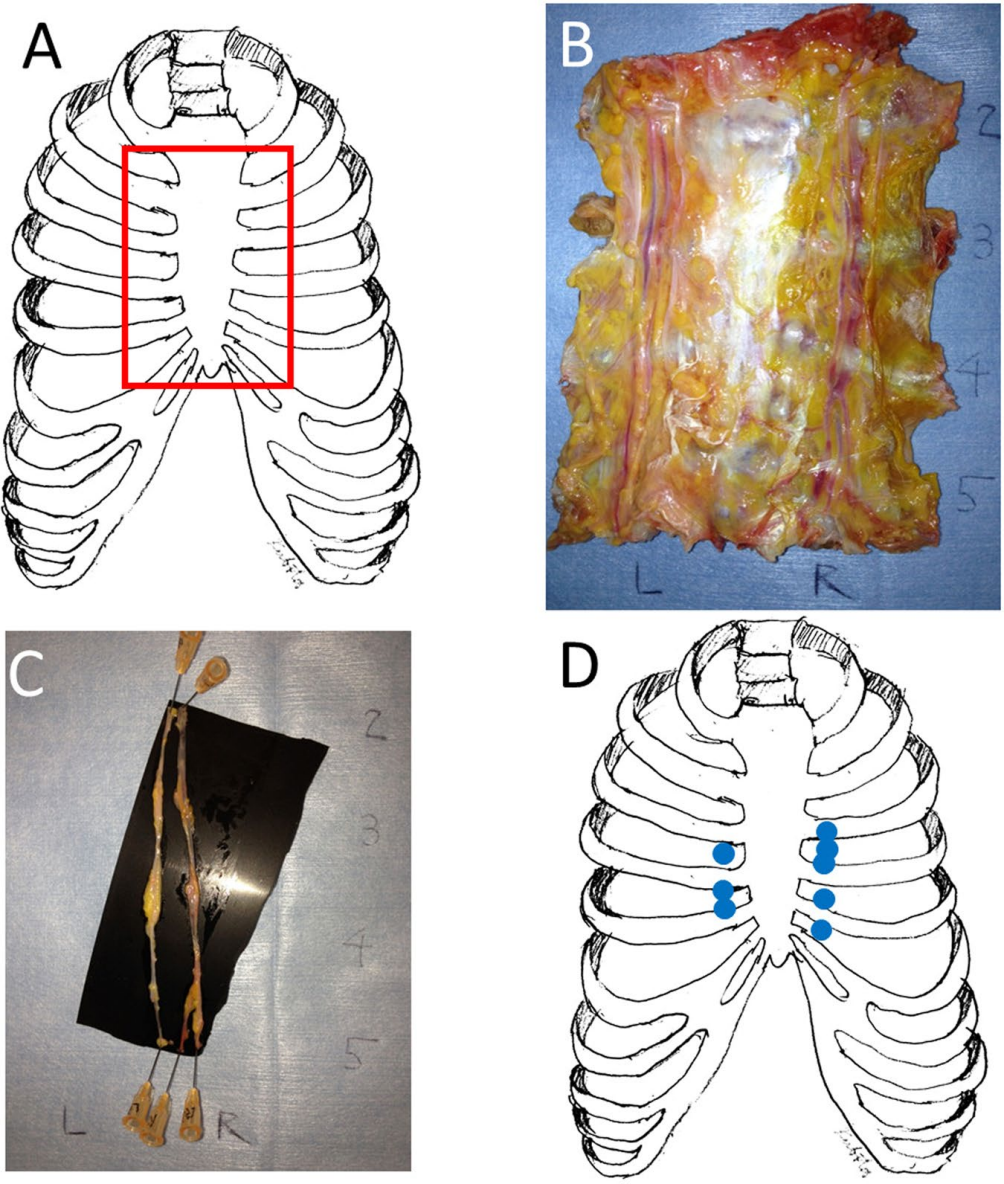
Internal mammary vein (IMV) samples. (**A**) A red line shows the area of sample taken from a fresh frozen cadaver. The complex taken en bloc from the chest consisted of the sternum, costal cartilages, intercostal muscles, parietal pleura, and IMVs. The sample complex could be easily harvested using the layer between the parietal pleura and visceral pleura. (**B**) The reverse side of the sample complex. IMVs can be clearly seen. (**C**) Obtained IMVs. IMV samples were pinned onto a rubber board with slight tension. (**D**) Locations of the IMV valves found in the study. Valves were found in 8 of 20 IMVs examined. No valves were found in the first and second intercostal spaces.

The IMV samples were collected through en bloc resection of a complex that consisted of the sternum, costal cartilages, intercostal muscles, parietal pleura, and bilateral IMVs from 10 human fresh frozen cadavers (Fig. 1). The area of samples taken was from below the inferior border of the first rib to the superior border of the sixth rib (Fig. 1A). IMVs were isolated from the underside surface of the samples (Fig. 1B). Isolated IMVs were stretched straight and pinned on a rubber board with 25-gauge needles placed on the proximal and distal ends of the vein (Fig. 1C). Then, the IMVs were immersed in 10% buffered formalin (Fujifilm Wako Pure Chemical, Osaka, Japan) for 24–72 hours. Next, the IMV samples were embedded in a paraffin block. The formalin-fixed, paraffin-embedded samples were cut perpendicular to the long axis of the IMVs into three or four blocks to fit onto glass slides. Then, the blocks were cut parallel to the long axis of the IMVs. Hematoxylin-eosin and Elastica van Gieson staining were performed. Venous valve structures were microscopically observed and recorded using a digital microscope BZ-9000 (Keyence, Osaka, Japan). As a control, the small saphenous vein (SSV) at the ankle level was also obtained. Hematoxylin-eosin staining of longitudinal sections of formalin-fixed, paraffin-embedded samples was performed in a similar way to the IMVs.

## Results

### Fresh frozen cadavers

Twenty IMVs were collected from 10 fresh frozen cadavers. Cadavers consisted of 5 females and 5 males. All cadavers were adult. Information on age and cause of death could not be obtained for this research study due to regulations. No cadaver had evidence of surgical intervention on his or her chest. The SSV was collected from one male fresh frozen cadaver as a control.

### Small Saphenous Vein (SSV) valve

**Figure 2:**
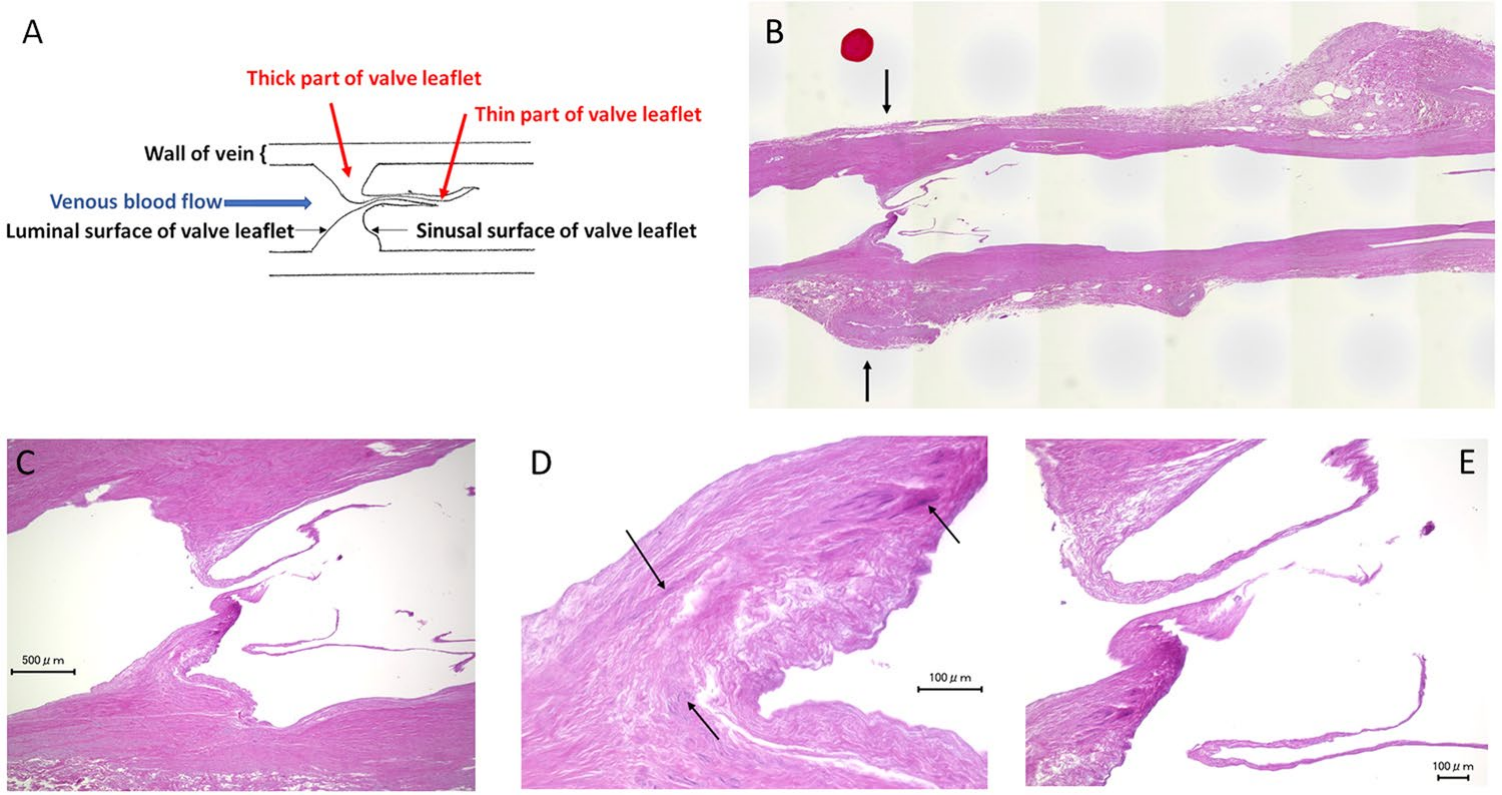
A venous valve in the small saphenous vein (SSV) at the ankle level. (**A**) Structure and names of the venous valve. (**B**) Overview of the SSV shows a robust structure of the valve (arrows). Longitudinal section. Hematoxylin-eosin staining. (**C**) Weak magnification view of the saphenous valve shows that the thick part of the valve leaflet can be clearly distinguished from the thin part of the valve leaflet. The height of the thick part of the valve leaflet nearly reaches the radius of the venous lumen. (**D**) Strong magnification view shows rich smooth muscle cells in the thick part of the valve leaflet (arrows). (**E**) In contrast, the thin part of the valve contains few smooth muscle cells.

Microscopic examination of the SSV showed robust valve structure (Fig. 2). The structure of the valve leaflet consisted of two parts (Fig. 2A,B,C). Thick and thin parts of the valve leaflet were clearly distinguishable. The “thick part of the valve leaflet” was located near the wall of the vein and consisted of smooth muscle cells and fibers, whereas the “thin part of the valve leaflet” was located near the center of the venous lumen and lacked smooth muscle cells (Fig. 2D,E). In the SSV, the height of the thick part of the valve leaflet reached close to the center of the venous lumen. In addition, the thick part of valve leaflet on one side of vein was close to the thick part of the valve leaflet on the opposite side.

### Internal Mammary Vein (IMV) valve

The microscopic examination showed venous valves in 6 (3 females and 3 males) of the 10 cadavers examined. There were valves in 8 of 20 IMVs examined. In the eight IMVs that had venous valves, there were three right IMVs and five left IMVs. In the seven of eight IMVs that had venous valves, there was only one valve. In one IMV obtained from a male cadaver, there were two valves. Two male cadavers had bilateral IMV valves. Most valves were found between the third and fifth costal cartilages (Fig. 1D). With respect to the female cadavers, IMV valves existed in three of five female cadavers. There were valves in 3 of 10 IMVs (1 right IMV valve and 2 left IMV valves). All three IMVs with valves had only one valve each. There were no cadavers with bilateral IMV valves. It was difficult to demonstrate a histologic difference in the valves between females and males in our study.

**Figure 3:**
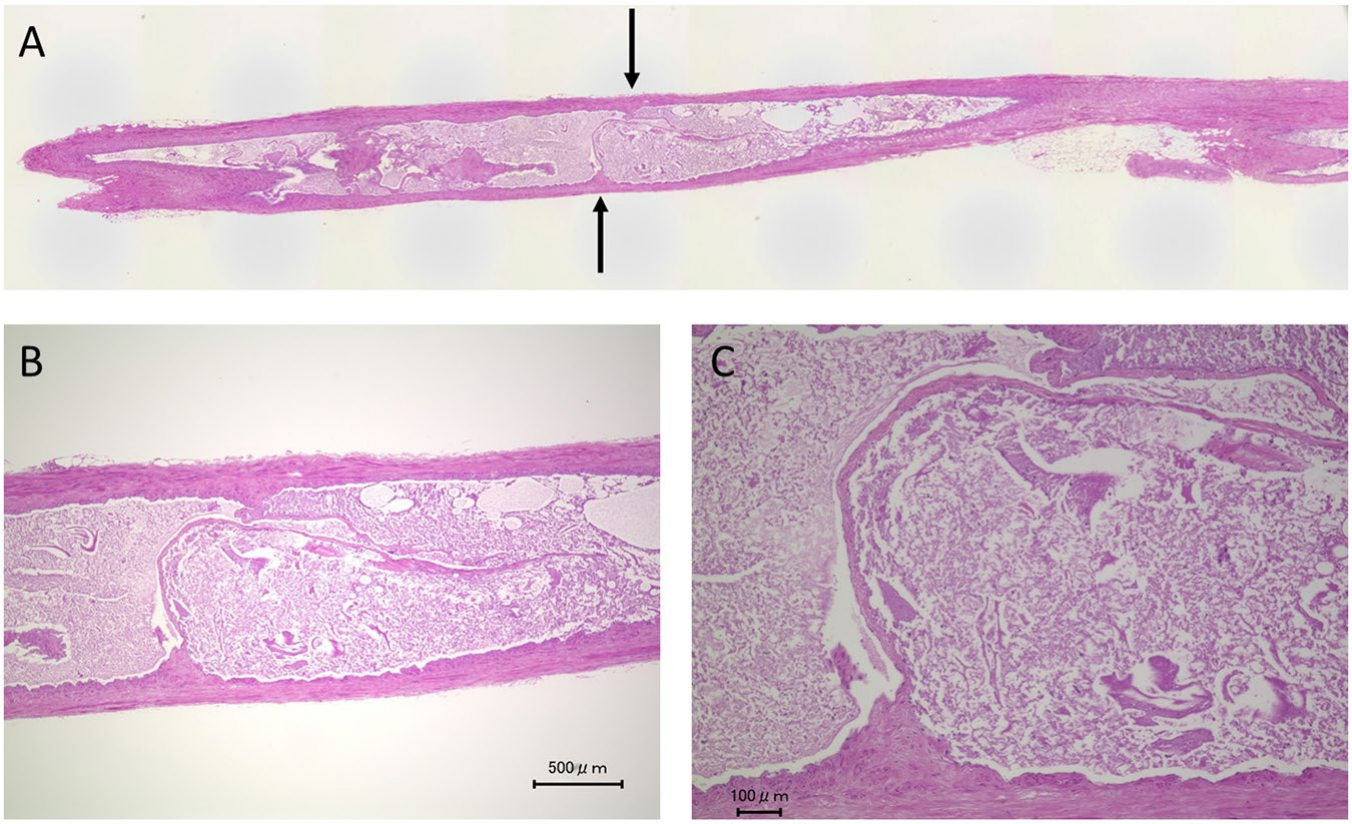
An internal mammary vein (IMV) valve. Longitudinal section. Hematoxylin-eosin staining. (**A**) Overview of the vein. The structure of the valve leaflet is less robust than that of the small saphenous vein (SSV) valve. (**B,C**) Weak and strong magnification views of the valve. The thick part of the IMV valve leaflet is smaller than that of the SSV valve leaflet. In contrast, there are no obvious differences in the size and structure of the thin part of the valve leaflet between the IMV and SSV.

**Figure 4:**
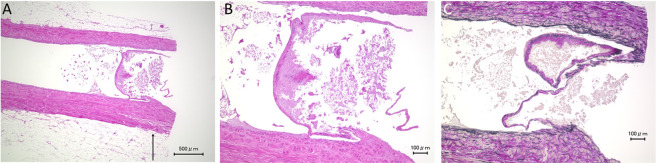
Other internal mammary vein (IMV) valve. Longitudinal section. (**A,B**) Hematoxylin-eosin staining. Weak magnification and strong magnification views show that the thick part of the valve is smaller than that of small saphenous vein valve. (**C**) Elastica van Gieson staining shows that the valve consisted of yellow-stained smooth muscle cells, black-stained elastic fibers and red-stained collagen fibers. The thick part of the valve leaflet contains yellow-stained smooth muscle cells, whereas there are no smooth muscle cells in the thin part of the valve leaflet.

**Figure 5:**
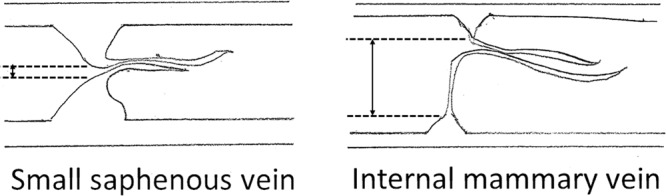
Schematic drawing of valves of the small saphenous vein (SSV) and the internal mammary vein (IMV). Size of the thick part of the IMV is smaller than the SSV. A gap between the thick part of the valve leaflet and the thick part of the opposite valve leaflet is wider in the IMV valve than the SSV valve (double-headed arrows). In contrast to the differences in shape and size of the thick part of the valve leaflet, the size and structure of the thin part of valve leaflet are similar between the SSV and IMV.

The size of the thick part of the valve leaflet in the IMV was smaller than the SSV, whereas there were no apparent differences in the size of the thin part of the valve leaflet between the IMV and SSV (Figs. 3 and 4). In the IMV valve, due to the low height of the thick part of the valve leaflet, there was a gap between opposite sides of the thick part of the valve leaflets (Fig. 5). Elastica van Gieson staining showed that the valve leaflets consisted of yellow-stained smooth muscle cells, black-stained elastic fibers, and red-stained collagen fibers (Fig. 4C). There were smooth muscle cells in the thick part of the valve leaflet in the IMV. The number of smooth muscle cells in the thick part of the valve leaflet in the IMV was smaller than the SSV. Similar to the thin part of the valve leaflet in the SSV, few smooth cells were observed in the thin part of the valve leaflet in the IMV.

## Discussion

This is the first study to show the existence of IMV valves microscopically, and we described the structural differences between IMV and SSV valves.

The question of whether venous valves exist or not in the IMV has been discussed in previous reports ^2–7,9–18^. The presence or absence of IMV valves has clinical importance in breast reconstructive surgery using autologous free tissue transfer with microsurgery. In free flap breast reconstruction, the retrograde IMV is used in some situations to secure venous drainage of the flap. If there was a valve in the IMV, venous drainage flow from the transplanted tissue to the retrograde limb of the IMV in the reverse flow direction would be jeopardized. According to some reports, the IMV is valveless. Kerr-Valentic *et al*., who first reported the use of retrograde anastomosis to the IMV, hypothesized that IMVs are valveless and retrograde anastomosis is safe^2^. A number of reports using the retrograde limb of IMV have accepted the valveless IMV hypothesis without any evidence. Only three clinical reports discussed the risk of IMV valves, if they exist, for retrograde anastomosis ^13,15,17^.

Before our study, there were only two reports of an anatomical study of IMV valves. Those two reports were non-histological studies, and the results were contradictory ^6,7^. There was one positive report by Mackey *et al*. in 2011 that showed the presence of IMV valves based on macroscopic exploration of the IMV in 32 formaldehyde-preserved cadavers^7^. There was also a negative report by Al-Dhamin *et al*. in 2014 that showed the IMV is valveless based on the observation of IMVs in six fresh frozen cadavers using stereoscopic surgical microscopy with a magnifying power of six^6^. We showed that the IMV has a valve by microscopic histological study of longitudinal sections of the IMV from 10 fresh frozen cadavers. Our results are consistent with the findings of Mackey *et al*. and inconsistent with the findings of Al-Dhamin *et al*. We think that our method is definitive to reject the valveless IMV hypothesis.

Our findings are consistent with the clinical results which dispute the valveless IMV hypothesis. We previously reported that in spite of the intraoperative patency of the retrograde limb of an IMV, postoperative occlusion of the retrograde limb of the IMV occurred in two of five (40%) cases confirmed by surgical exploration or color Doppler imaging, and the possible presence of IMV valves in these patients might be one of the reasons^13^. Sugawara *et al*.^15^ reported that flow into the retrograde IMV anastomosis evaluated by intraoperative indocyanine green angiography was absent in 11 of 40 cases (28%), and the possible presence of IMV valves in these patients might be one of the reasons. A venous valve in the IMV shown in our study could account for the difficulty with venous blood flow into the retrograde limb of the IMV. However, the effect of an IMV valve on venous flow into the retrograde limb of the IMV is still unclear. Al-Dhamin *et al*. reported that manually injected methylene blue into a retrograde limb of an IMV in fresh cadavers flowed smoothly in all 10 IMVs that were examined^6^.

Few reports are available on the histological structure of venous valves^19,20^. Our study is the first to show histological images of IMV valves. In humans, most studies on venous valves were performed on valves in the lower extremities^21–25^. Our study showed that the size of the thick part of the valve leaflet of the IMV was smaller than that of the SSV. In contrast, there were no obvious differences in the structure and size of the thin part of the valve leaflet between the IMV and SSV. Histologically, venous valves consist of collagen fibers, elastic fibers, smooth muscles cells, and covering endothelium^18^. Smooth muscle cells exist only in the thick part of the valve leaflet. The smooth muscle layer of the thick part of the valve leaflet is continuous with the smooth muscle cell layer of the vein wall^19^. Collagen fibers and elastic fibers are abundant in both the thick and thin parts of the valve leaflet. We think that the smaller size of the thick part of the valve leaflet in the IMV compared with the SSV is due to a smaller amount of smooth muscle cells in the thick part of the valve leaflet. We also think that the paucity of smooth muscle cells in the thick part of the IMV valve leaflet is a result of low hydrostatic pressure in the IMV. In a standing position, hydrostatic pressure increases by gravitational effect at a rate of 0.77 mmHg/cm of vertical displacement from the cephalad to caudal direction^26^. Hydrostatic pressure in the SSV at ankle level in the standing position is roughly 100 mmHg higher than that in the IMV.

It is unclear whether IMV valve regurgitation can occur. Judging from rather common valvular dysfunction in the lower extremities, we think that IMV valve regurgitation can occur in certain circumstances. Tomioka *et al*. reported that venous blood pressure in the flap vein was 10–30 mmHg higher than that in the retrograde limb of the IMV in some cases^3^. Our study suggested that a smaller thick part of the valve leaflet in the IMV than the SSV can lead to more backflow across the IMV than the SSV valve.

Our study showed that less than half of the IMVs that we examined had valves. Judging from the small-sized thick part of the valve leaflet in the IMV and low frequency of IMV valves in our study population, we think there is a possibility that the IMV valve is a rudimentary structure that has little physiological significance. The gap between the thick part of the valve leaflet and the thick part of the opposite valve leaflet in the IMV shown in our study suggests that IMV valve has little or no ability to prevent regurgitation. Further study is needed to prove the presence of IMV valve regurgitation and to evaluate the value of free flap transfer using retrograde anastomosis.

## Conclusions

Our histological study of human IMVs showed that the IMV is not valveless. The size of the valve leaflet in the IMV was smaller than that in the SSV.
